# Synthesis, crystallographic characterization, molecular docking and biological activity of isoquinoline derivatives

**DOI:** 10.1186/s13065-017-0321-1

**Published:** 2017-10-16

**Authors:** Hatem A. Abuelizz, Rashad Al-Salahi, Jamil Al-Asri, Jérémie Mortier, Mohamed Marzouk, Essam Ezzeldin, Azza A. Ali, Mona G. Khalil, Gerhard Wolber, Hazem A. Ghabbour, Abdulrahman A. Almehizia, Gehad A. Abdel Jaleel

**Affiliations:** 1 Department of Pharmaceutical Chemistry, College of Pharmacy, King Saud University, P.O. Box 2457, Riyadh, 11451 Saudi Arabia; 2 Department of Pharmaceutical & Medicinal Chemistry, Institute of Pharmacy, Freie Universität Berlin, Königin-Luise Str. 2-4, 14195 Berlin, Germany; 3 Department of Chemistry, College of Science and Humanities, Prince Sattam bin Abdulaziz University, 83, Alkharj, Saudi Arabia; 4 Chemistry of Natural Products Group, Center of Excellence for Advanced Sciences, National Research Centre, Dokki, Cairo, 12622 Egypt; 5 Drug Bioavailability Lab., College of Pharmacy, King Saud University, P.O. Box 2457, Riyadh, 11451 Saudi Arabia; 6 Department of Pharmacology & Toxicology, Faculty of Pharmacy, Al-Azhar University, Cairo, Egypt; 7 Department of Pharmacology & Toxicology, Faculty of Pharmacy, Modern University for Technology and Information, Cairo, Egypt; 8 Department of Pharmacology , National Research Centre, El-Bohoth St., Dokki, Cairo, 12622 Egypt

## Abstract

The main objective of this work was to synthesize novel compounds with a benzo[*de*][1,2,4]triazolo[5,1-*a*]isoquinoline scaffold by employing (dioxo-benzo[*de*]isoquinolin-2-yl) thiourea as a building block. Molecular docking was conducted in the COX-2 active site to predict the plausible binding mode and rationalize the structure–activity relationship of the synthesized compounds. The structures of the synthesized compounds were confirmed by HREI-MS, and NMR spectra along with X-ray diffraction were collected for products **1** and **5**. Thereafter, anti-inflammatory effect of molecules **1**–**20** was evaluated in vivo using carrageenan-induced paw edema method, revealing significant inhibition potency in albino rats with an activity comparable to that of the standard drugs indomethacin. Compounds **8**, **9**, **15** and **16** showed the highest anti-inflammatory activity. However, thermal sensitivity-hot plat test, a radiological examination and motor coordination assessment were performed to test the activity against rheumatoid arthritis. The obtained results indicate promising anti-arthritic activity for compounds **9** and **15** as significant reduction of the serum level of interleukin-1β [IL-1β], cyclooxygenase-2 [COX-2] and prostaglandin E2 [PGE2] was observed in CFA rats.

**Electronic supplementary material:**

The online version of this article (doi:10.1186/s13065-017-0321-1) contains supplementary material, which is available to authorized users.

## Introduction

Inflammation is an important defense mechanism against infective, chemical, and physical aggressions. Deregulation of this mechanism can lead to pathological perturbations in the body, as observed for example with allergies, autoimmune diseases and organ transplantation rejection [1]. A key modulator of the inflammatory response is prostaglandin E_2_ (PGE_2_), generated at the inflammation site from arachidonic acid via the cyclooxygenase (COX) enzyme [2].

Non-steroidal anti-inflammatory drugs (NSAIDs) are widely used against inflammation, as for example in the treatment of chronic and acute inflammation [3], pain management [4], and fever [5]. However, cardiovascular problems, gastrointestinal lesions and nephrotoxicity have been observed in case of long NSAIDs treatment [6]. Therefore, the discovery of novel anti-inflammatory drugs with less side effects remains an intensive area of research in medicinal chemistry. Two isoforms of the cyclooxygenase have been characterized: COX-1 and COX-2. COX-2 levels increase after inflammatory stimuli induced by mitogens or cytokines, and can be lowered by glucocorticoids [7]. Recent discovery indicates that renal toxicity and gastrointestinal side effects observed with NSAIDs can be due to COX-1 inhibition, while selective inhibition of COX-2 shows a comparable anti-inflammatory response with less side effects [8]. As an example, naproxen is a non-selective COX inhibitor, like oxicam, it belongs to a group of NSAID displaying mixed COX inhibition, characterized by slow, reversible, and weak inhibitor binding. Contrary to other NSAIDs that inhibit COX reversibly and rapidly (mefenamic acid and ibuprofen), or irreversibly and slowly (indomethacin and diclofenac), naproxen contributes to the cardioprotective effect because of their weak inhibition of COX [9].

The quinolone ring system is often found in synthetic compounds with various biological activities, including anti-convulsant [10], anti-malarial [11], anti-microbial [12], and anti-inflammatory [13] effects. Quinolines and their isomers isoquinolines are also found in various natural products, such as quinine (anti-malarial) and quinidine (anti-arrhythmic) [14]. Furthermore, many isoquinoline alkaloids, including cepharanthine, berberine and tetrandine, have shown anti-inflammatory effect [15]. The binding affinity and the solubility in physiological conditions can be considerably affected by the position of the nitrogen bearing a side chain on the isoquinoline skeleton [16]. Therefore, a huge effort has been spent in developing novel and effective isoquinoline derivatives. On the other hand, the triazole moiety is found in many important biologically active compounds. Synthesized molecules with a triazole moiety possess anti-tubercular [17], antimicrobial [18], anti-cancer [19] and anti-inflammatory [20] activity. Triazole-based heterocyclic derivatives have enhanced biological activity or possess new biological activities [21]. A wide range of triazole containing compounds are clinically used drugs and developed via molecular hybridization approach including anticancer, antifungal, antibacterial, antiviral, antitubercular, anti-inflammatory, antiparasitic, anticonvulsant, antihistaminic and other biological activities [21]. Therefore, triazole and isoquinoline can be promising chemical fragments for the design of novel anti-inflammatory drugs.

Considering the importance of the isoquinoline and triazole moieties in the wide range of anti-inflammatory treatments and its interesting activity profile, we conducted a molecular-hybridization design of novel anti-inflammatory compounds. Toward the development of new effective and selective anti-inflammatory agents, the [1,2,4]triazole and [5,1-*a*]isoquinoline were integrated to develop a novel class of inhibitor.

## Materials and methods

Melting points were determined on open glass capillaries using a STUART Melting point SMP 10 apparatus and are uncorrected. NMR spectra were recorded on a Bruker AMX 500 spectrometer in DMSO-*d*_*6*_ and reported as δ ppm values relative to TMS at 500/700 and 125/176 MHz for ^1^H and ^13^C NMR, respectively. *J* values were recorded in Hz. HREI-MS spectra were measured on a JEOL MStation JMS-700 system. X-ray data were collected on a Bruker APEX-II D8 Venture area diffractometer, equipped with graphite monochromatic Mo *K*α radiation, λ = 0.71073 Å at 100 (2) K. Follow-up of the reactions and checking the purity of compounds was made by TLC on DC-Mikrokarten polygram SIL G/UV_254_, from the Macherey–Nagel Firm, Duren Thickness: 0.25 mm.

### Procedure for preparation of 1-(1,3-dioxo-1*H*-benzo[*de*]isoquinolin-2(3*H*)-yl)thiourea (**1**)

To a solution of 1,8-naphthalic anhydride (2.2 mmol) in boiling glacial acetic acid (15 mL), thiosemicarbazide (2.8 mmol) was added and left the mixture stirring under reflux for 1 h. The obtained solid was separated and washed with water. Recrystallization by a mixture of toluene and DMF yielded the final product as colorless crystals (90%); mp 243–244 °C; ^1^H NMR (500 MHz, DMSO-*d*_*6*_): δ 9.82 (s, 1H, –NH–), 8.55 (br d, *J* = 8 Hz, 2H, H-3/8), 8.51 (br d, *J* = 8 Hz, 2H, H-5/6), 7.99 (br s, 2H, –NH_2_), 7.92 (t, *J* = 8 Hz, 2H, H-4/7); ^13^C NMR (125 MHz, DMSO-*d*_*6*_): δ 181.8 (C=S), 162.0 (C-2/9), 134.7 (C-5/6), 131.3 (C-5a), 130.9 (C-3/8), 127.3 (C-4/7), 122.7 (C-2a/8a), 119.0 (C-2b); HRMS (EI), *m/z* Calcd for C_13_H_9_N_3_O_2_S (M)^•+^ 271.0983, found 271.1013.

### Procedure for preparation of 10-(methylthio)-7*H*-benzo[*de*][1,2,4]triazolo[5,1-*a*]isoquinolin-7-one (**3**)

A mixture of **I** or **2** (1 mmol) with dimethyl-*N*-cyanoimidodithiocarbonate (1 mmol) in *N,N*-dimethyl formamide (10 mL) was refluxed in the presence of triethylamine for 4 h. Afterwards, the mixture was poured into ice/water, the resulting solid was filtered, washed with water and dried. Analytically pure product obtained as yellow amorphous powder (67%); mp 268–269 °C; ^1^H NMR (700 MHz, DMSO-*d*_*6*_): δ 8.75 (br d, *J* = 7.7 Hz, 1H, H-6), 8.61 (br d, *J* = 8.4 Hz, 1H, H-3), 8.59 (br d, *J* = 8.4 Hz, 1H, H-8), 8.47 (br d, *J* = 7.7 Hz, 1H, H-5), 7.99 (t, *J* = 7.7 Hz, 1H, H-4), 7.93 (t, *J* = 7.7 Hz, 1H, H-7), 2.73 (s, 3H, –*S*–CH_3_); ^13^C NMR (176 MHz, DMSO-*d*_*6*_): δ 166.0 (C-9), 156.6 (>*C*–S–CH_3_), 155.9 (C-2), 137.0 (C-6), 134.3 (C-3), 133.5 (C-5), 132.3 (C-8a), 128.4 (C-8), 128.1 (C-4), 128.0 (C-7), 126.1 (C-5a), 122.9 (C-2b), 118.2 (C-2a), 14.2 (*S*-CH_3_); EI-MS, *m/z* (%): 267 [(M^·+^, 100)]; HRMS (EI), *m/z* Calcd for C_14_H_9_N_3_OS (M)^·+^ 267.0466, found 267.0490.

### Procedure for preparation of 10-(methylsulfonyl)-7*H*-benzo[*de*][1,2,4]triazolo[5,1-*a*]isoquinolin-7-one (**5**)

An amount of **3** (1 mmol) was dissolved in boiling glacial acetic acid (12 mL), afterward H_2_O_2_ (12 mL), was added dropwise over a period of 10 min., while heating. After the addition was complete, the mixture was poured into hot water and left at room temperature, the obtained solid was collected, washed with water and dried. Recrystallization from DMF gave analytically pure colored as pale brown amorphous powder (60%); mp 218–219 °C; ^1^H NMR (500 MHz, DMSO-*d*_*6*_): δ 8.93 (br d, *J* = 7.5 Hz, 1H, H-6), 8.29 (br d, *J* = 8 Hz, 1H, H-3), 8.26 (br d, *J* = 8 Hz, 1H, H-8), 8.23 (br d, *J* = 7.5 Hz, 1H, H-5), 7.78 (t, *J* = 8 Hz, 1H, H-4), 7.67 (t, *J* = 8 Hz, 1H, H-7), 3.47 (s, 3H, –*SO*_2_–CH_3_); ^13^C NMR (125 MHz, DMSO-*d*_*6*_): δ 165.9 (C-9), 161.3 (>C–S–CH_3_), 160.4 (C-2), 136.0 (C-6), 135.9 (C-3), 131.9 (C-5), 131.7 (C-8a), 128.4 (C-8), 128.3 (C-4), 127.6 (C-7), 127.4 (C-5a), 117.3 (C-2b), 112.7 (C-2a), 42.6 (–*SO*_2_–CH_3_); HRMS (EI), *m/z* Calcd for C_14_H_9_N_3_O_3_S (M)^•+^ 299.1296, found 299.1316.

### Procedure for preparation of 10-(phenoxy)-7*H*-benzo[*de*][1,2,4]triazolo[5,1-*a*]isoquinolin-7-one (**4**)

A mixture of **I** or **2** (1 mmol) with diphenoxy-*N*-cyanoimidocarbonate (1 mmol) in *N,N*-dimethyl formamide (10 mL) was refluxed in the presence of triethylamine for 4–5 h. Afterwards, the mixture was poured into ice/water, the obtained solid was filtered, washed with water and dried. Analytically pure product resulted as brown amorphous powder (45%); mp 281–282 °C; ^1^H NMR (500 MHz, DMSO-*d*_*6*_): δ 8.68 (br d, *J* = 7.5 Hz, 1H, H-6), 8.45 (br d, *J* = 8 Hz, 1H, H-3), 8.31 (br d, *J* = 8 Hz, 1H, H-8), 8.01 (br d, *J* = 7.5 Hz, 1H, H-5), 7.86 (t, *J* = 8 Hz, 1H, H-4), 7.78 (t, *J* = 8 Hz, 1H, H-7), 7.48 (dt, *J* = 8.5, 1 Hz, 2H, H-3′/5′), 7.28 (dd, *J* = 8.5, 1 Hz, 2H, H-2′/6′), 7.16 (br t, *J* = 8 Hz, 1H, H-4′); ^13^C NMR (125 MHz, DMSO-*d*_*6*_): δ 167.1 (C-OPh), 165.8 (C-9), 155.6 (C-2), 150.5 (C-1′), 136.9 (C-6), 135.1 (C-3), 133.1 (C-5), 132.7 (C-8a), 129.8 (C-3′/5′), 128.6 (C-8), 128.2 (C-4), 127.9 (C-7), 126.3 (C-5a), 123.8 (C-4′), 122.5 (C-2b), 119.2 (C-2′/6′), 118.6 (C-2a); HRMS (EI), *m/z* Calcd for C_19_H_11_N_3_O_2_ (M)^•+^ 313.1296, found 313.1310.

### Procedure for preparation of 8-hydrazinocarbonyl-1-naphthoic acid (**6**)

A solution of **2** (1 mmol) in DMF (10 mL) was refluxed with Conc. HCl (15 mL) for 24 h. The mixture poured into ice/water, the obtained solid was separated, washed with water and dried. Analytically pure product resulted as yellow powder (60%); mp 225–226 °C; ^1^H NMR (500 MHz, DMSO-*d*_*6*_): δ 14.30 (s, 1H, –COOH), 8.97 (br s, 3H, –NH–NH_2_), 8.56 (br d, *J* = 7.5 Hz, 1H, H-2), 8.48 (br d, *J* = 7.5 Hz, 1H, H-4), 8.39 (br d, *J* = 7.5 Hz, 1H, H-5), 8.32 (br d, *J* = 7.5 Hz, 1H, H-7), 7.91 (t, *J* = 8 Hz, 1H, H-3), 7.85 (t, *J* = 8 Hz, 1H, H-6); ^13^C NMR (125 MHz, DMSO-*d*_*6*_): δ 166.4 (–*C*OOH), 158.9 (–*C*ONH–NH_2_), 135.7 (C-2), 134.9 (C-4), 132.4 (C-5), 131.9 (C-4a), 131.7 (C-8), 131.7 (C-7), 127.3 (C-6), 124.6 (C-3), 123.8 (C-8a), 117.7 (C-1); HRMS (EI), *m/z* Calcd for C_12_H_10_N_2_O_3_ (M)^•+^ 230.0691, found 230.0709.

### Animals

Adult albino rats weighing 130–150 g were obtained from the animal house colony in the National Research Centre (Giza, Egypt). Animals were subjected to controlled conditions of temperature (25 ± 3 °C), humidity (50–60%) and illumination (12-h light, 12-h dark cycle, lights on at 08:00 h) and were provided with standard pellet diet and water ad libitum for 1 week before starting the experiment.

### Anti-inflammatory activity

**Fig. 1:**
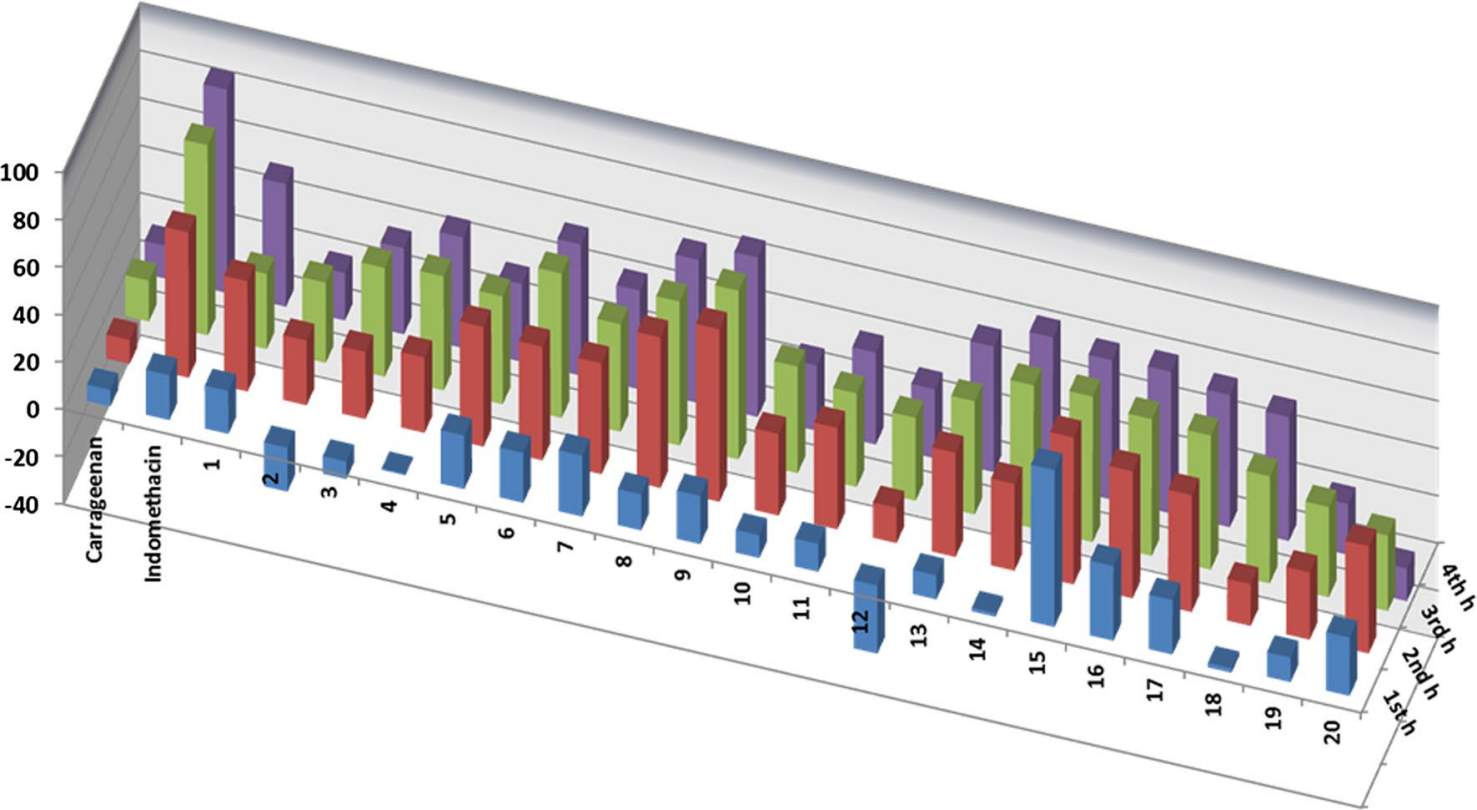
Reduction of rat’s paw edema induced by carrageenan after administration of tested compounds

The anti-inflammatory effect was evaluated in correspondence to the carrageenan-induced paw edema method [22]. Briefly, carrageenan (1% w/v, 0.1 mL/paw) was injected into right hind paw at the plantar side. Rats were observed for abnormal behavior and physical condition after carrageenan injection. The right paw was measured once before (normal baseline) and then after carrageenan injection at 1, 2, 3, and 4 h. Twenty groups of female Sprague–Dawley rats were used (n = 6, weighing 130–150 g). According to the procedure reported in the literature, the first group represented the control carrageenan injected, the second was given indomethacin (Sigma, USA) orally, the reference anti-inflammatory drug (10 mg/kg) [23], and the remaining groups were treated with the tested compounds (25 mg/kg bodyweight) orally, 1 h before carrageenan (Sigma, USA) injection. Paw volume was measure by using a water displacement plethysmometer (UGO BASILE 21025 COMERIO, ITALY). The percentage increase in paw volume was calculated using (Oedema volume of test/baseline volume) * 100 − 100. Moreover, percentage (%) inhibition was calculated using (1 − D/C) × 100, where, D-represents the percentage difference in increased paw volume after the administration of test drugs to the rats. C-represents the percentage difference of increased volume in the control groups Fig. 1.

### Anti-arthritic activity

#### Induction of arthritis and treatment protocol

Adjuvant arthritis (AA) was induced in female Wistar rats by subcutaneous (SC) injection of 0.1 mL CFA (Sigma-Aldrich, USA) into the plantar surface of the right hind paw, which exhibit**s** many similarities to human RA. The severity of the induced adjuvant disease was followed by measurement of the volume of the injected paw by using a water displacement plethysmometer (UGO Basile 21025, Comerio, Italy). The paw volume of the injected right paw over vehicle control is measured at every week during experiment [24]. Rats were randomly divided into four groups of six rats each: normal control, untreated arthritis group, compound **9** treated, and compound **15** treated arthritis groups. Results were expressed as the percentage increase in paw volume.

#### Thermal sensitivity hotplate test

Rats were placed on the hotplate at 55 °C, one at a time (Columbus Instruments, Columbus, OH). The latency period for hind limb response (e.g. shaking, jumping, or licking) was recorded as response time. Each trial had a maximum time of 45 s. The rat was removed from the hotplate immediately after a response was observed [25].

#### Motor coordination assessment methods for RA

Motor coordination and balance was assessed using a rota rod apparatus (Med Associates, Italy) [26, 27]. All rats underwent a 3-day training program, by which time a steady baseline level of performance was attained. During that period, rats were trained to walk against the motion of a rotating drum at a constant speed of 12 rpm for a maximum of 2 m. In total, four training trials per day with an interval trial time of 1 h were performed. Rats falling off during a training trial were put back on the rotating drum. Following the training days, a 1 day test of three trials was performed using an accelerating speed levels (4–40 rpm) over 5 min. The apparatus was wiped with a 70% ethanol solution and dried before each trial. The mean latency to fall off the rotarod was recorded, and rats remaining on the drum for more than 300 s were removed and their time scored as 300 s.

#### Radiographic assessment of arthritis in rat paws

Radiographic assessment was used blindly at end of the disease to evaluate the severity of OA radiography, using an X-ray collimator, model R-19, lamp-type 24 V, 90 W, on-load voltage 19 V (Ac max KVP 100 KVP min. inh, filt. 1 m, Japan). At the end of the experiment, 24 h after the last dose of treatment, blood samples were collected under light anaesthesia with diethyl ether by puncturing rato-orbital plexus; the blood was allowed to flow into a dry, clean centrifuge tube and left to stand 30 m before centrifugation to avoid haemolysis. Then, blood samples were centrifuged for 15 m at 2500 rpm, and the clear supernatant serum was separated and collected by Pasteur pipette into a dry, clean tube to use for determination of the serum levels of PGE2, COX2 and IL-1β [28].

#### Statistical analysis

Data were expressed as mean ± SEM and analysed by one-way analysis of variance (ANOVA) for multiple comparisons followed by Tukey’s post hoc comparisons. All analyses were performed by SPSS statistics package version 17.0 (SPSS, Chicago, IL, USA). P value of ≤0.05 was considered statistically significant.

#### Molecular modelling

All compounds were prepared using MOE (Molecular Operating Environment, 2011) and CORINA [29, 30]. Murine cyclooxygenase-2 (COX-2) enzymes co-crystallized with indomethacin (PDB entry 4COX [31, 39]) and co-crystallized with naproxen (PDB entry 3NT1 [32, 34]) were used as templates for the modeling study. Since indomethacin and naproxen have different interactions with COX-2, both inhibitors were used as references in this study [32].

The software GOLD version 5.2 [33] was used to perform docking. The crystal structure depicted under PDB entries 4COX and 3NT1 were first protonated, and water molecules as well as co-crystallized ligands were deleted before docking. In this study, default parameters were used with no constraints (binding site: within 10 Å around the co-crystallized ligand, scoring functions: GOLDScore, genetic algorithm: 100% search efficiency). Validation of the docking protocol was performed by recovering the original conformation of the co-crystallized inhibitor inside the active site. The root mean square deviation (RMSD) between the docked pose and the crystal structure of 0.7 Å was measured for indomethacin, and 0.25 Å for naproxen (Fig. 5).

The followed strategy in this work was to generate ten docking poses for each compound using GOLD, and compare them to the one of the two co-crystallized inhibitors. This state-of-the-art approach developed in our group has been applied and validated in various recent studies [34–37]. Using the software LigandScout 3.1 [35], a 3D pharmacophore model that represents the steric interactions of the co-crystallized inhibitor inside the COX-2 pocket was created (Fig. 5) and used as a scoring function to analyze the resulting docking poses.

All generated docking poses were minimized with the MMFF94 force field inside the COX-2 pocket using LigandScout 3.1 [35]. The 3D-pharmacophore of indomethacin and the quality of the superposition of each pose with the co-crystallized ligand were used to prioritize the poses that could best explain the biological behaviors of the studied molecule. Those only were used for comparing and discussing inhibitors binding modes. LigandScout was also used for analysis, pharmacophore creation, and visualization.

#### Crystal structure determination for compound **5** (CCDC 1049988)

Yellow needle-shaped crystals of crystal structure determination for compound XX of C_14_ H_9_ N_3_ O_3_ S_1_ are, at 293 (2) K Monoclinic, space group *P*21/*n*, with a = 13.7295 (10) Å, b = 12.0853 (10) Å, c = 16.2631 (12) Å, β = 114.859 (2)°, V = 2448.4 (3) Å^3^ and Z = 7 formula units [d_calcd_ = 1.624 Mg/m^3^; µ (MoKα) = 0.28 mm^−1^]. A full hemisphere of diffracted intensities was measured using graphite monochromated MoK radiation (=0.71073 Å) on a Bruker SMART APEXII D8 Venture Single Crystal Diffraction System. X-rays were provided. The Bruker software package SHELXTL [36] was used to solve the structure using “direct methods” techniques. All stages of weighted full-matrix least-squares refinement were conducted using F_o_^2^ data with the SHELXTL software package.

The final structural model incorporated anisotropic thermal parameters for all non hydrogen atoms and isotropic thermal parameters for all hydrogen atoms. The remaining hydrogen atoms were included in the structural model as fixed atoms (using idealized sp^2^- or sp^3^-hybridized geometry and C–H bond lengths of 0.95–0.98 Å) “riding” on their respective carbon atoms. The isotropic thermal parameters for these hydrogen atoms were fixed at a value 1.2 (non-methyl) or 1.5 (methyl) times the equivalent isotropic thermal parameter of the carbon atom to which they are covalently bonded. A total of 369 parameters were refined using no restraints and 2 data. Final agreement factors at convergence are: R_1_ (unweighted, based on *F*) = 0.096 for 4307 independent “observed” reflections having 2θ (MoKα)< 50.0° and I > 2(I); wR_2_(weighted, based on F^2^) = 0.264 for all 2979 independent reflections having 2θ (MoKα)< 50.0°.

## Results

### Chemistry

**Scheme 1:**
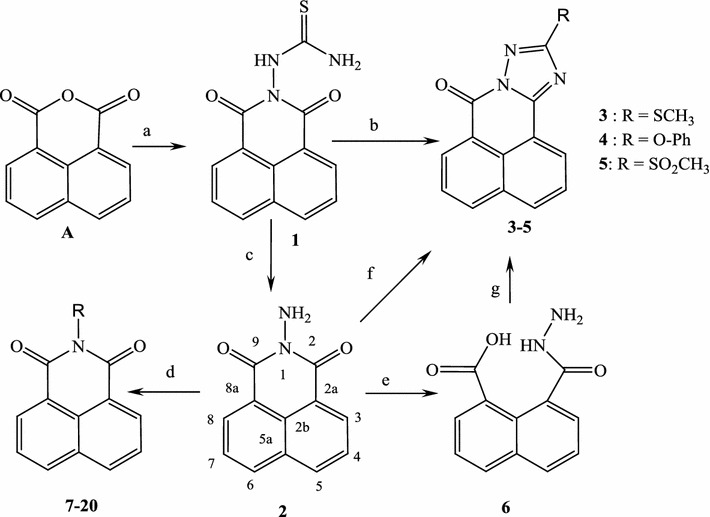
Synthetic routes for products **1**‒**20**.* a* Thiosemicarbazide, glacial acetic acid, reflux;* b* dimethyl-*N*-cyanoimido(dithio)carbonate, diphenoxy-*N*-cyanoimi -docarbonate, Et3N, DMF, H2O2, galcial acetic acid, reflux;* c* NaOH, NH2NH2, HCl, DMF, refux;* d* aldehydes, isothiocyanates, acetic anhydrides, DMF, glacial acetic acid, reflux;* e* HCl, DMF, reflux;* f* dimethyl-*N*-cyanoimido(dithio)carbonate, diphenoxy-*N*-cyanoimidocarbonate, Et3N, DMF, H2O2, galcial acetic acid, reflux;* g* dimethyl-*N*-cyanoimido(dithio)carbonate, diphenoxy-*N*-cyanoimidocarbonate, Et3N, DMF, reflux

**Fig. 2:**
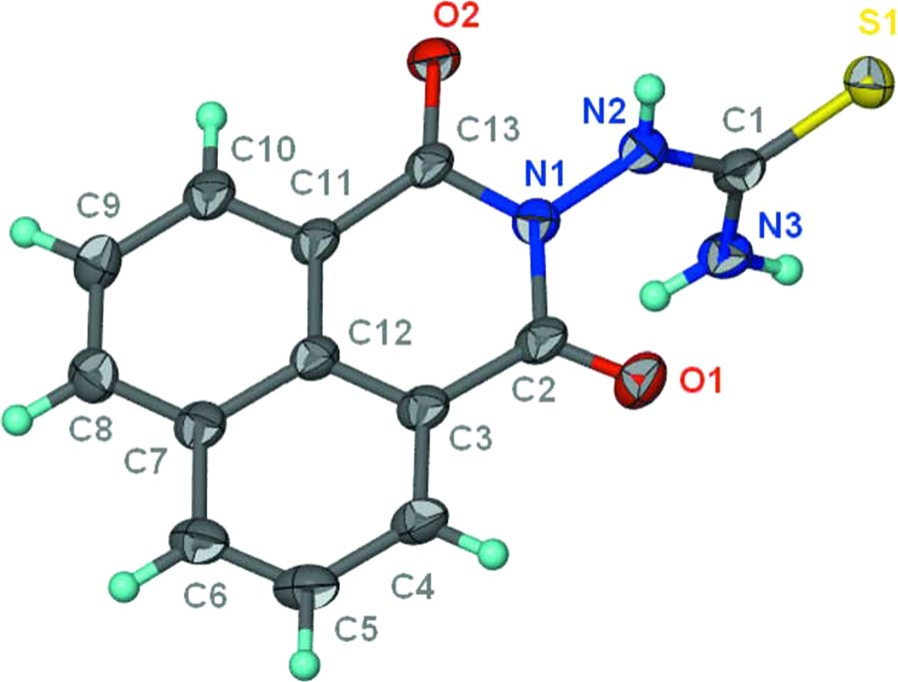
ORTEP diagram of compound **1**

**Fig. 3:**
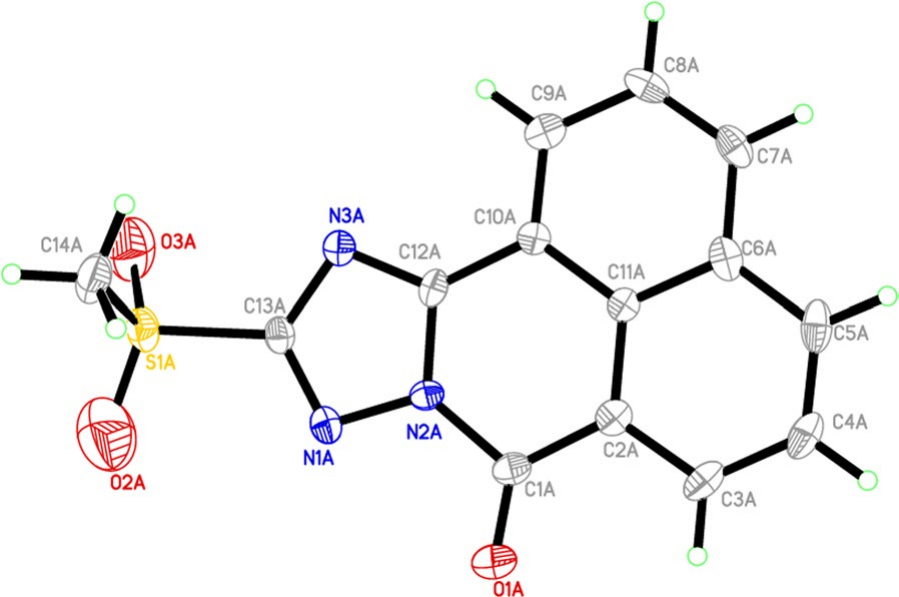
ORTEP diagram of compound **5**

As dioxo-benzo[*de*]isoquinolin-2-yl)thiourea (**1)** was required as key starting material (see Scheme 1), it was previously prepared by the reaction of 1,8-naphthalic anhydride (**A**) with thiosemicarbazide. This compound was then characterized by X-ray crystallography (Figs. 2, 3) [37]. The symmetric structure of the 2-amino-1*H*-benzo[*de*]isoquinolin-1,3-dione moiety in **1** shows similar NMR splitting patterns and δ-values (δ_H_ and δ_C_) to **A**, including three pairs of two equivalent aromatic protons (H-3/8, H-5/6 and H-4/7) and their corresponding ^13^C-signals. Presence of the thio-urea moiety was supported by the –NH and –NH_2_ singlets at δ_H_ 9.82 and 7.99, respectively alongside C=S carbon at 181.8 in ^1^H and ^13^C NMR spectra. Finally, HREI-MS confirmed the identity of **1** through a molecular ion peak (M)^•+^ at *m/z* 271.1013 calculated for 271.0983 and a MF of C_13_H_9_N_3_O_2_S. Reaction of **1** with hydrazine hydrate in presence of NaOH produced product **2** in good yields (78%). Structure of **2** was confirmed by ^13^C-NMR analysis, which showed the disappearance of C=S at 181.80 ppm. Based on the high reactivity of **1** towards hydrazine, it was anticipated that **1** would react with *N*-cyanoimido(dithio)carbonates in a similar manner in presence of Et_3_N to give novel benzo[*de*][1,2,4]triazolo[5,1-*a*]isoquinolines **3** and **4**. Further oxidation of methylthio in **3** using H_2_O_2_ yielded in the novel benzo[*de*][1,2,4]triazolo[5,1-*a*]isoquinoline **5**. Similarly, treatment of **2** or **6** with *N*-cyanoimido(dithio)carbonates with basic medium, resulted in compounds **3** and **4**, respectively (Scheme 1).

Ring-closure of products **3**–**5** was reflected into the deformation of the symmetrical structures of the 2-aminoisoquinoline moiety, which appeared in the ^1^H NMR spectra as four broad doublets (H-3, 5, 6 and 8) and two triplicates (H-4, 7) signals. Also, ring-closure of a fused triazole ring was confirmed from the characteristic δ values of C-2 (≈156–160 ppm), carbonyl-carbon (C-9) at about δ 166 ppm and the methythio-, methylsulfonyl- and phenoxyl-bearing carbon signals at 156.6, 161.3 and 167.1, respectively. ^1^H NMR of **3** and **5** showed a characteristic singlet of methylthio and methylsulfonyl at δ 2.73 and 3.74 together with their carbons at 14.2 and 42.6 ppm, respectively, to prove the insertion of such functional groups. The phenoxyl group in the structure of **4** was concluded through its characteristic resonances at 7.48 (dt, *J* = 8.5), 7.28 (dd, *J* = 8.5) and 7.16 (br t, *J* = 8), attributable for H-3′/5′, H-2′/6′, and H-4′, and their C-signals at δ 129.8, 119.2, and 123.8, respectively. The success of the previous reactions was finally proven by the unambiguous confirmation of the 3D-structure of methylsulfonyl product **5** by X-ray crystallography (Figs. 2, 3). The open structure **6** was obtained by heating compound **2** with concentrated HCl in DMF for 24 h under reflux (Scheme 1). This structure was confirmed by the two singlets at δ 14.30 and 8.97 ppm, interpretable for –COOH and –NH–NH_2_ protons, together with the corresponding carbonyl ^13^C-resonances at δ 166.4 and 158.9, for –COOH and –CONHNH_2_, respectively. Compounds **7**–**20** were synthesized from 2-amino-1*H*-benzo[*de*]isoquinolin-1,3-dione (**2**) (Table 1) and reported in our previous work [38].

**Table 1:**
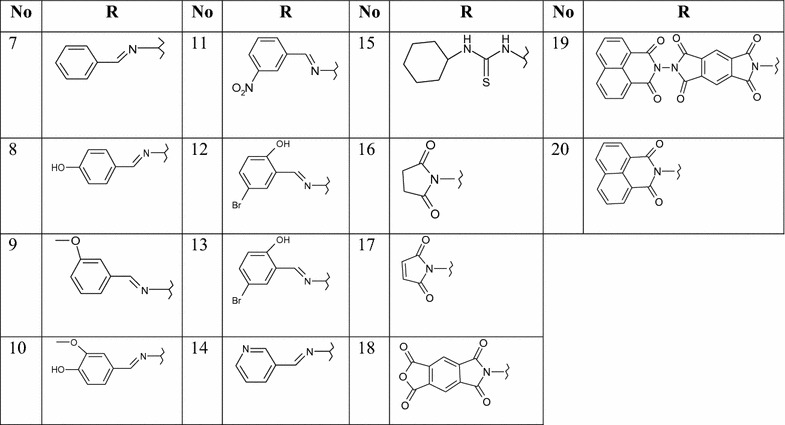
Synthesized compounds **1**‒**20**

### Anti-inflammatory activity

In-vivo anti-inflammatory effects of the synthesized benzo[*de*]isoquinolines **1**–**20** were then evaluated using standard carrageenan-induced paw edema in rats, indomethacin as reference drug. Paw swelling is good index for evaluating and assessing the degree of inflammation and the therapeutic and curative effects of bioactive compounds. The response of target compounds **1**–**20** ranged from weak to moderate activity, however, some of compounds exhibited promising effects in a direct correlation with their structural variation. In comparison to the control and reference drugs, all investigated compounds show significant reduction of paw size throughout a 4 h time period (Additional file 1: Table S1; Fig. 1).

### Anti-arthritic activity

**Fig. 4:**
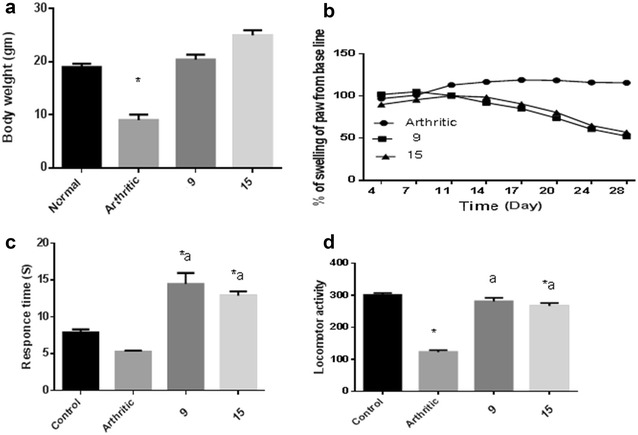
**a** Changes in body weight of CFA-induced RA in rat with compound**s 9** and **15**. **b** Effect of test compounds (50 mg/kg) on CFA-induced arthritis in rats with compounds **9** and **15**. **c** Effect on hotplate time response with compounds **9** and **15**. **d** Effect on spontaneous motor activity in CFA rats with compounds **9** and **15**. Data represent the mean ± SEM (n = 6 for each group); *significance versus control (P ≤ 0.05); ^a^significance versus CFA group (P ≤ 0.05)

In chronic inflammation, CFA-induced arthritic model is considered the best known experimental model of rheumatoid arthritis (RA) and a model of chronic polyarthritis with features that resemble RA. Basis on the promising anti-inflammatory activity results of compounds **9** and **15**, we extended our research to evaluate their anti-arthritic effects in doses of 50 mg/kg administered orally. For monitoring the progression of arthritis in a CFA-induced albino rat model, a number of assessment methods as thermal sensitivity hotplate, motor coordination and radiographic were applied. The changes in the body weight of the CFA-induced arthritis in rat with compounds was measured (Fig. 4a). Measurement of paws was performed by using a plethysmometer (Fig. 4b). The sensitivity and reaction to pain stimulus was indicated by hotplate (Fig. 4c). The serum level of Interleukin-1β (IL-1β, cyclooxygenase-2 (COX2) and prostaglandin E2 (PGE2) of the CFA treated rat were measured and compared to the control group (Table 2). Soft tissue with normal bone density of the rat’s hind paws was examined by X-ray (Additional file 1: Figure S1A).

**Table 2:** Effect of test compounds 50 mg/kg on serum IL-1β, COX2 and PGE2 of rat CFA

	IL-1β (pg/mL)	COX2 (ng/mL)	PGE2 (pg/mL)
Control (vehicle)	26.8 ± 1.4	15.9 ± 0.4	17.3 ± 0.9
CFA	93 ± 2.2^a^	46.3 ± 1.5^a^	65.9 ± 2.3^a^
Compound **9**	37.4 ± 1.1^a,b^	16.9 ± 0.7^b^	22.6 ± 0.7^b^
Compound **15**	41.9 ± 0.9^a,b^	20.8 ± 0.5^a,b^	25.4 ± 0.3^a,b^

Data represent the mean ± SEM (n = 6 for each group)

^a^Significance versus control (P ≤ 0.05)

^b^Significance versus CFA group (P ≤ 0.05)

### Molecular modelling

**Fig. 5:**
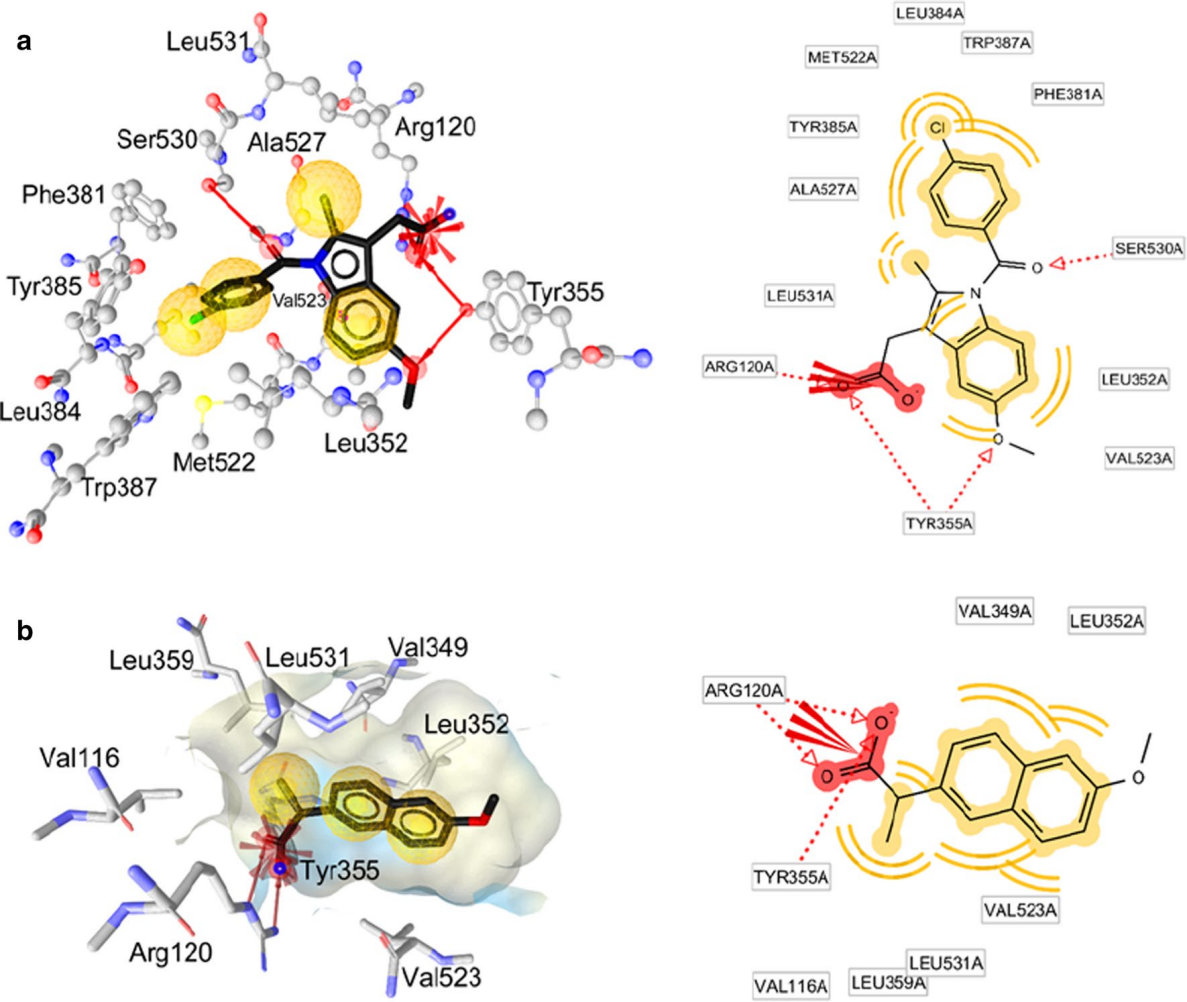
**a** Binding mode of indomethacin co-crystallized with COX-2 as 3D (left) and 2D (right), PDB entry 4COX. **b** Binding mode of naproxen co-crystallized inside COX-2 active site as 3D (left) and 2D (right), PDB entry 3NT1. Pharmacophore features created using LigandScout. Red arrows: H-bonds, red star: negative ionizable feature, yellow spheres: hydrophobic contacts

With the aim to predict the most plausible binding mode of the identified inhibitors in this work and to rationalize their structure–activity relationship (SAR), molecular docking was performed in the COX-2 active site. To investigate the inhibition of these synthesized compounds, ten docking conformations were generated, carefully analyzed and prioritized using a 3D-pharmacophore representation of the binding modes of the reference inhibitors, indomethacin and naproxen. Since the sequence of murine COX-2 active site is 87% identical to the one in human, PDB entry 4COX of murine COX-2 co-crystallized with indomethacin, and PDB entry 3NT1 co-crystallized with naproxen were selected for this computer-aided study [39]. The docking program GOLD 5.2 [33] was used to reproduce the binding mode of the co-crystallized indomethacin in the ligand–protein complex 4COX [39] and the co-crystallized naproxen inside the complex 3NT1 [32]. The root mean square deviation (RMSD) between the heavy atoms of the original co-crystallized ligand, and the docked conformation ligand was calculated in GOLD 5.2. Validation of docking experiments for the PDB codes 4COX and 3NT1 for COX-2 enzyme are depicted in Additional file 1: Figure S2. The co-crystal of indomethacin in the COX-2 active site (PDB entry 4COX) and naproxen inside the COX-2 active site (PDB entry 3NT1) were analyzed (Fig. 5a, b).

Docking results were evaluated by MolDock score function and hydrogen bond and hydrophobic interactions between tested compounds and the target receptor were used to compare between the tested compounds and the reference compounds (Table 3).

**Table 3:** MolDock scores of tested compounds

Ligand	MolDock score	Ligand	MolDock score
1	−99.3452	11	−114.071
2	−98.9549	12	−109.498
3	−107.022	13	−111.854
4	−121.189	14	−103.154
5	−68.9738	*15*	*−128.385*
6	−92.9481	16	−94.8682
7	−108.334	17	−98.9912
8	−110.781	*18*	*−128.489*
9	−106.802	19	−104.212
10	−120.592	20	−71.3571
Indomethacin	*−151.314*		

### Docking with indomethacin as reference inhibitor

Firstly, software LigandScout [35] was used to analyze molecular interactions of indomethacin and naproxen in the COX-2 active site. Three kinds of interactions can be identified for indomethacin inside COX-2 active site: hydrophobic contacts between aromatic rings of indomethacin and hydrophobic residues Phe381, Leu384, Met522, Tyr385, Trp387, Leu531, Leu352, Ala527 and Val523 in the active site, a salt bridge formed between Arg120 and the carboxylate group of the inhibitor, and hydrogen bonds between the inhibitor and Tyr355, Arg120, and Ser530. The original conformation of indomethacin in the crystal structure 4COX was used as a reference for investigating and prioritizing the generated docking poses. The resulting conformations of the studied compounds were analyzed and the most plausible poses were selected based on their ability to create similar interactions as the one of the reference inhibitor.

**Fig. 6:**
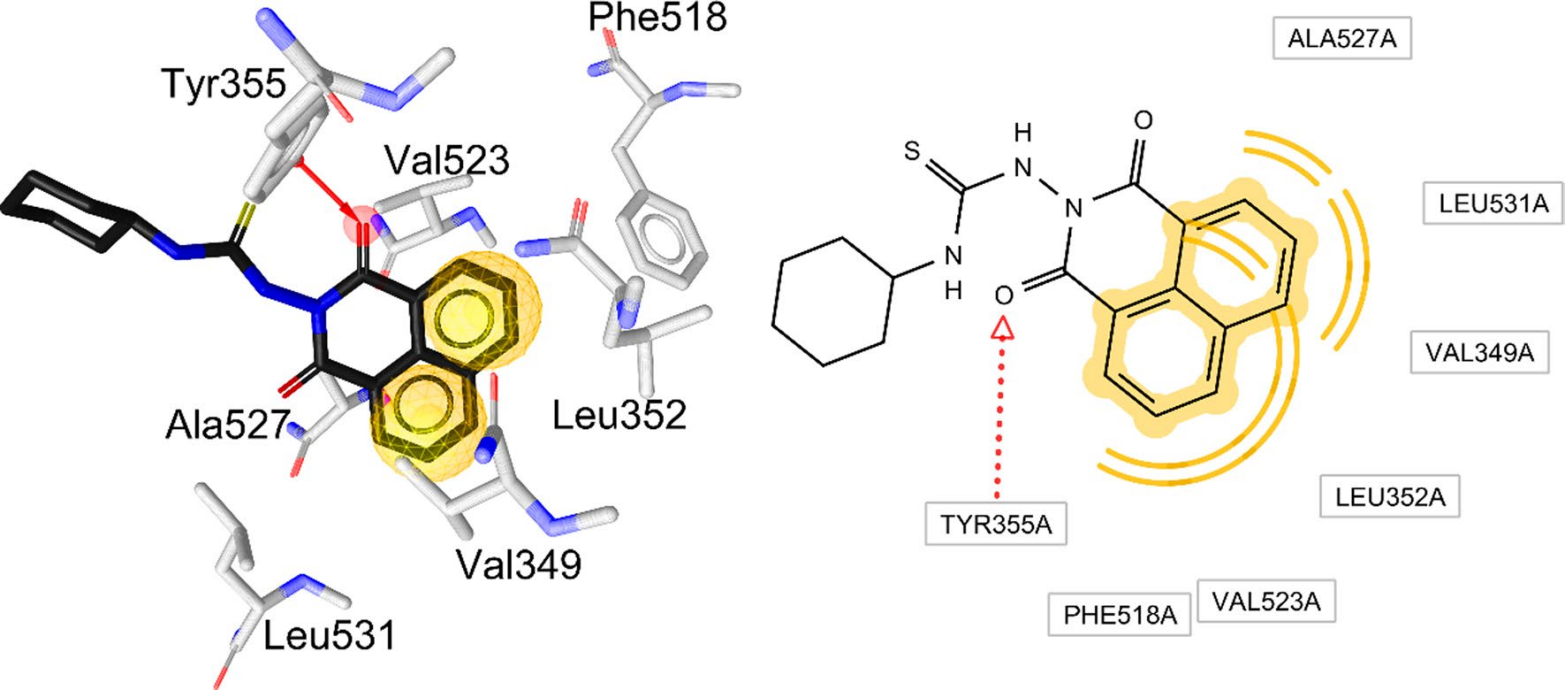
The predicted binding mode of **15** in the COX-2 pocket (PDB: 4COX). Above: 3D (left) and 2D (right). Yellow spheres denote hydrophobic contacts. Red arrow represents hydrogen bond acceptor

Docking of compound **15** reveals an ability to partially accommodate the same region as the reference inhibitor inside the pocket (Fig. 6). Compound **15** shows binding through hydrophobic contacts formed between its fused aromatic rings and Val523 and Val349, as observed for indomethacin. Additional hydrophobic contacts can be formed between the fused aromatic rings and Ala527, Leu531, Leu352, and Phe518. One hydrogen bond can be formed between the carbonyl oxygen of 15 and Tyr355. Superposition of 15 with indomethacin shows how both ligands can occupy the same region in the pocket, which can explain why **15** is the most potent inhibitor in this series (Fig. 6).

### Docking with naproxen as reference inhibitor

Naproxen is another reference inhibitor that is stabilized in the active site of COX-2 with a different binding mode compared to indomethacin. It is important to investigate the ability of our compounds to interact with COX-2 using a binding mode more similar to the one of naproxen as the one of indomethacin. Therefore, the generated binding conformations of our inhibitor series was aligned to a 3D-pharmacophore model extracted from the COX-2-naproxen co-crystal (PDB entry 3NT1) [32]. The ability of these molecules to fulfill similar interactions as the one identified in this crystal structure was analyzed using LigandScout.

**Fig. 7:**
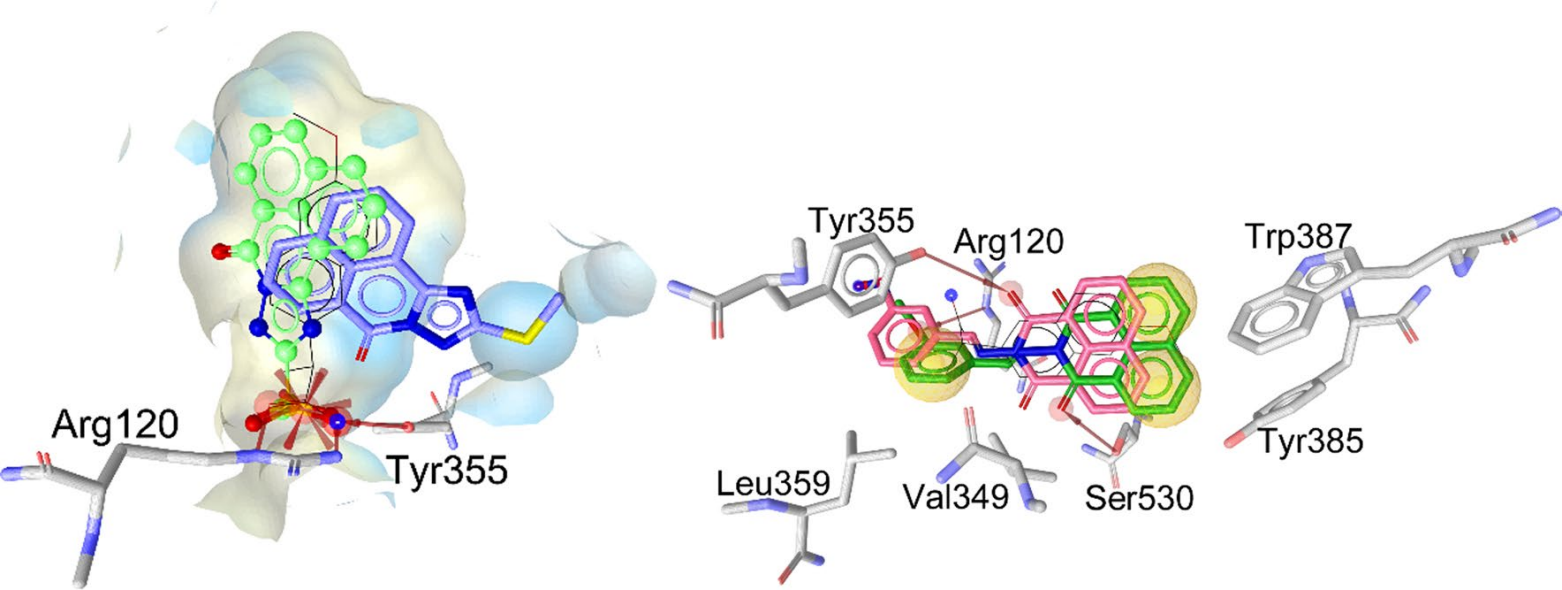
Left: predicted binding modes of **2** (blue sticks), and **3** (green balls and sticks) superimposed with original conformation of naproxen (black lines) inside the COX-2 active site (PDB entry: 3NT1). Red arrows represent hydrogen bonds. Right: suggested binding modes for **9** (green sticks) superposed with the original conformation of naproxen (black lines) inside the COX-2 active site (PDB entry: 3NT1)

The plausible binding mode of **9**, the most potent inhibitor in this series, shows interesting interactions with the binding site of COX-2. Besides the stabilization of ligands inside the pocket with hydrophobic contacts, hydrogen bonds can be formed between the methoxy of compound **9** (up to 72%) and Arg120, and a carbonyl of **9** with Ser530 (Fig. 7). This interaction may serve to anchor the compound within the active site similar to naproxen and enforce the binding orientation.

## Discussion

The results revealed that many of the tested compounds caused significant decrease in paw edema after 1, 2, 3, 4 h from drug administration. The edema inhibition percentages measurements show that after 1 h compounds **2**, **3**, **12**, **14** and **18** were inactive. The same result was observed after 4 h with compound **20**. Compounds **4**, **10, 11, 13,** and **19** showed low activity (0.63, 9.60, 11.75, 10.12 and 10.36% of inhibition, respectively) 1 h after drug administration, while **12** and **18** showed 14.89 and 17.60% of inhibition after 2 h. 1 h after drug administration, compounds **5**–**7**, **9**, **16**, **17** and **20** were found to possess a good biological response (from 20.61 to 31.45%) compared to indomethacin (18.98%), and compound **15** emerged as the most potent (66.13%), 3.5 more than reference indomethacin (Additional file 1: Table S1). With respect to the effect of indomethacin after 2 h, a similar tendency were observed for compounds **8** and **15**; **1**, **5**–**8**, **11**, **13**, **16**, **17** and **20** showed a comparable good activity; a moderate effect was observed by **2**–**4**, **10**, **14** and **19,** however, compound **9** showed a stronger effect (72.72%) than indomethacin (61.22%). Similarly, after 3 h, compounds **6**, **8**, **9** and **14**–**17** showed good activity (56.55–71.09%); and moderate inhibition was observed for compounds **1**–**5**, **7**, **10**–**13**, **19** and **20** (31.72**–**48.09%), while indomethacin inhibited 80.15% of the induced edema. 4 h after drug administration, compounds **1**, **6**, **8**, **9**, and **13**–**18** exhibited remarkable and significant activity (51.67–67.20%) compared to indomethacin (85.70%); a moderate effect was observed for compounds **3**–**5**, **7** and **11** (32.81–46.66%), and compounds **2**, **10**, **12** and **19** showed the lowest potency (Fig. 1).

Transformation of **1** into **2**–**4** attenuated the anti-inflammatory effect. However, subsequently transformation of **2** and **3** into **6** and **5** respectively, was offered advantageous influencing in the terms of activity. Opening the bulky-sized structure **2** into **6** is crucial for inducing the anti-inflammatory effects. Various effects were recorded upon chemical transformation of **2** into **7**–**20**, particularly compounds **8**, **9** and **14** that appeared to be the most active products among aldehyde derivatives **7**–**14**. This could be attributed to the presence of hydroxyl group on benzyl ring in the structure of **8**, and methoxy group in **9** which they could be displayed an essential role in activity. However, the presence of a hetero atom in **14** does seem to offer remarkable advantage for activity. This indicates that increasing the number of hetero atoms that can act as hydrogen bond donor could be responsible for the improvement of anti-inflammatory activity. Incorporation of a crucial structural feature of cyclohexyl isothiocyanate in **2** to afford **15** increased the anti-inflammatory activity. Furthermore, an improvement in the activity was observed by insertion of succinic anhydride function in the structure of **2** to give **16**.

In regards to chronic inflammation, compound **9** and **15** have demonstrated a significant effect on the CFA-induced arthritic model. When compounds **9** and **15** were administered daily for 3 weeks (starting from day 7 of the CFA induction); non-significant change in body weight was noticed, however a significantly reduction (52%) on the body weight of the CFA-induced RA group was observed in comparison to the control group (Fig. 4a). As illustrated in Fig. 4b, an adjuvant injection resulted in an acute inflammatory phase of swelling in the injected paws for 3 days and an autoimmune phase of swelling in both injected and non-injected paws after 10 days. Compounds **9** and **15** markedly reduced the volumes of injected paws in comparison to the CFA (RA) group.

The sensitivity and reaction to pain stimulus was indicated by hotplate, to which the response of the CFA group rats was slower than the control group. Treatment of the CFA rats with compounds **9** and **15** normalized the response compared to the control group (Fig. 4c), significantly altered the total number of movements (P ≤ 0.05) in the locomotor activity at the end of the experiment (Fig. 4d), and reduced the serum level of Interleukin-1β (IL-1β, cyclooxygenase-2 (COX2) and prostaglandin E2 (PGE2) compared to the control group (Table 2).

Examination of the rats hind paws by X-ray showed normal soft tissue with normal bone density (Additional file 1: Figure S1A). Aggravated swelling with bone erosion in the articular facet and joint space that was practically devastated was observed after 28 days from CFA induction (Additional file 1: Figure S1B). Treatment with compounds **9** and **15** showed increasing swelling of soft paw tissue with a diminution in bone density (Additional file 1: Figure S1C) and some minimal soft tissue swelling (Additional file 1: Figure S1D) respectively.

Kinetic, mutagenesis, structure–activity relationship analysis and x-ray crystallography studies of naproxen and indomethacin elucidated the molecular determinants for COX inhibition [32]. Because a detailed comparison of the binding mode of these two drugs could unveil the responsible features for selectivity, analysis of the enzyme-inhibitor interaction for indomethacin and naproxen co-crystallized with COX-2 was conducted using a pharmacophore approach with software LigandScout [35]. Compounds **1**–**20** have MolDock scores range from −68.9 to −128.4. Compounds **18** and **15** which have highest MolDock score in this experiment 128.4 and 128.3, respectively. Compound **15** which has the best percentage of edema inhibition in this study give also best MolDock score (Table 3). The co-crystal of indomethacin in the COX-2 active site (PDB entry 4COX) shows three critical interactions: (i) hydrophobic contacts between aromatic rings of indomethacin and hydrophobic residues Phe381, Leu384, Met522, Tyr385, Trp387, Leu531, Leu352, Ala527 and Val523 in the active site (ii) a salt bridge formed between Arg120 and the carboxylate group of the inhibitor, and (iii) hydrogen bonds between the inhibitor and Tyr355, Arg120, and Ser530 (Fig. 5a). Analysis of naproxen inside the COX-2 active site (PDB entry 3NT1) indicates a binding mode similar from the one of indomethacin. The key interactions with Arg120 and Tyr355 with the carboxylate group of the ligand interactions are conserved, and most hydrophobic contacts observed in the main pocket are accommodating the lipophilic moieties of the inhibitor. The only difference between the two inhibitors is the chlorobenzoyl group of indomethacin that reaches another region of the active site (Tyr385, Leu384, Phe381) that naproxen cannot reach because of its smaller size (Fig. 5b).

The investigation of docking conformations for the most potent inhibitors in this series indicates the important role of hydrophobic contacts to stabilize ligands inside the pocket. However, the lack of interaction with the critical Tyr385 with most of these series implies their moderate/weak potencies against COX-2. Interestingly, Compound **15** shows fast inhibitory potency (66%) from the first hour and remains relatively stable even after 4 h (58%). Thus, it is worth to investigate this series using a reference such as naproxen that has different binding mode compared to indomethacin. The binding conformation of compounds **15** and **9** forming hydrogen bonds with Tyr355 in similar manner to naproxen confirms the importance of Tyr355 and confirms the findings highlighted with indomethacin. Also, the unique formation of hydrogen bond between Arg120 and naproxen can be observed with compounds **9** and **15**. This result suggests that the most potent inhibitor in this series, compound **15**, has a binding mode that is highly correlated with the conformation of naproxen inside COX-2 active site. Moreover, compound **9** can interact through Leu352, Val349, Ser530, Trp387, Tyr385 and its carbonyl group with Tyr355 by hydrogen bonding. These interactions can explain that compound **9** is higher potency than compound **15** (up to 66%), which lacks these interactions.

By using a combined approach of biological activity and molecular modeling, we were able to probe the importance of **9** and **15**-COX-2 interactions and elucidate key interactions and compare it with indomethacin and naproxen. The combination of mutagenesis and structural studies will clearly define the contribution of protein and inhibitor atoms to affinity with compound **9** and **15**, which will be applied in the future.

## Conclusion

The present investigation explored the significance of the molecular-hybridization development of novel compounds with strong activity against the inflammation-induced-by-carrageenan model. Compounds **8**, **9**, **15** and **16** were the most potent compounds of the series, which can be explained by a similar binding mode to references indomethacin and naproxen. Moreover, the results presented here support the notion that compound **9** and **15** are active against rheumatoid arthritis and significantly reduce the serum level of Interleukin-1β [IL-1β], cyclooxygenase-2 [COX2] and prostaglandin E2 [PGE2] in the CFA rats. The investigation of the inhibitor in the COX-2 binding site by molecular modeling confirms the importance of the hydrogen bonds formation with Tyr355 and Arg120 and its role in the potency of the newly synthesized inhibitors. Furthermore, the hydrophobic contacts formed by compounds **9** and **15** with Tyr385 suggest binding modes that are highly correlated with the conformation of naproxen and indomethacin inside COX-2 active site. These compounds offer a base for further investigation of a novel anti-inflammatory and anti-arthritic agents.

## Additional file

**Additional file 1: Table S1.** Reduction of rats paw edema induced by carrageenan after administration of tested compounds. **Figure S1.** Radiological characteristics of hind paws of arthritic rats. **Figure S2.** Software GOLD 5.2 generated binding mode of indomethacin (left) and naproxen (right) compared to their original co-crystalized conformations. Left: generated binding mode of indomethacin (blue, balls and sticks) in the PDB: 4COX compared to its experimental conformation (black sticks). Right: created binding mode of naproxen (pink, balls and sticks) in the PDB: 3NT1 compared to its co-crystal conformation (black sticks).

## Abbreviations

ORTEP: Oak Ridge Thermal-Ellipsoid Plot Program
[IL-1β]: interleukin-1
COX: cyclooxygenase
PGE2: prostaglandin E2
