# Supplementary material for: Synthesis, crystallographic characterization, molecular docking and biological activity of isoquinoline derivatives
Source: Chem Cent J. 2017 Oct 16;11:103. doi: 10.1186/s13065-017-0321-1 (PMC5643734; doi:10.1186/s13065-017-0321-1)
Supplement: Supplementary file 1 — Additional file 1: Table S1. Reduction of rats paw edema induced by carrageenan after administration of tested compounds. Figure S1. Radiological characteristics of hind paws of arthritic rats. Figure S2. Software GOLD 5.2 generated binding mode of indomethacin (left) and naproxen (right) compared to their original co-crystalized conformations. Left: generated binding mode of indomethacin (blue, balls and sticks) in the PDB: 4COX compared to its experimental conformation (black sticks). Right: created binding mode of naproxen (pink, balls and sticks) in the PDB: 3NT1 compared to its co-crystal conformation (black sticks). [file 13065_2017_321_MOESM1_ESM.docx]

**Table S1.** Reduction of rats paw edema induced by carrageenan after administration of tested compounds.

| **Treatment Compound** | **% Reduction** | | | |
| --- | --- | --- | --- | --- |
|  | **1^st^ H** | **2^nd^ H** | **3^rd^ H** | **4^th^ H** |
| Carrageenan | 6.92 | 10.13 | 17.27 | 13.81 |
| Indomethacin | 18.98 | 61.22 | 80.15 | 85.70 |
| **1** | 18.12 | 46.51 | 31.72 | 51.67 |
| **2** | -18.53 | 27.29 | 33.77 | 20.02 |
| **3** | -7.44 | 28.30 | 45.83 | 36.28 |
| **4** | 0.63 | 31.78 | 48.09 | 46.66 |
| **5** | 22.56 | 50.45 | 45.47 | 32.81 |
| **6** | 21.05 | 47.94 | 60.87 | 55.25 |
| **7** | 25.73 | 46.70 | 45.30 | 41.66 |
| **8** | 15.20 | 63.49 | 60.69 | 60.35 |
| **9** | 20.38 | 72.72 | 71.09 | 67.20 |
| **10** | 9.60 | 34.37 | 44.92 | 27.49 |
| **11** | 11.75 | 42.72 | 39.44 | 38.76 |
| **12** | -28.72 | 14.89 | 34.91 | 29.11 |
| **13** | 10.12 | 43.87 | 47.74 | 52.94 |
| **14** | -1.51 | 36.59 | 60.25 | 62.61 |
| **15** | 66.13 | 61.95 | 61.32 | 58.80 |
| **16** | 31.45 | 53.53 | 57.65 | 59.68 |
| **17** | 23.01 | 49.29 | 56.55 | 55.69 |
| **18** | -1.85 | 17.60 | 45.19 | 52.22 |
| **19** | 10.36 | 28.37 | 38.01 | 21.34 |
| **20** | 24.61 | 45.19 | 31.80 | -13.72 |

| 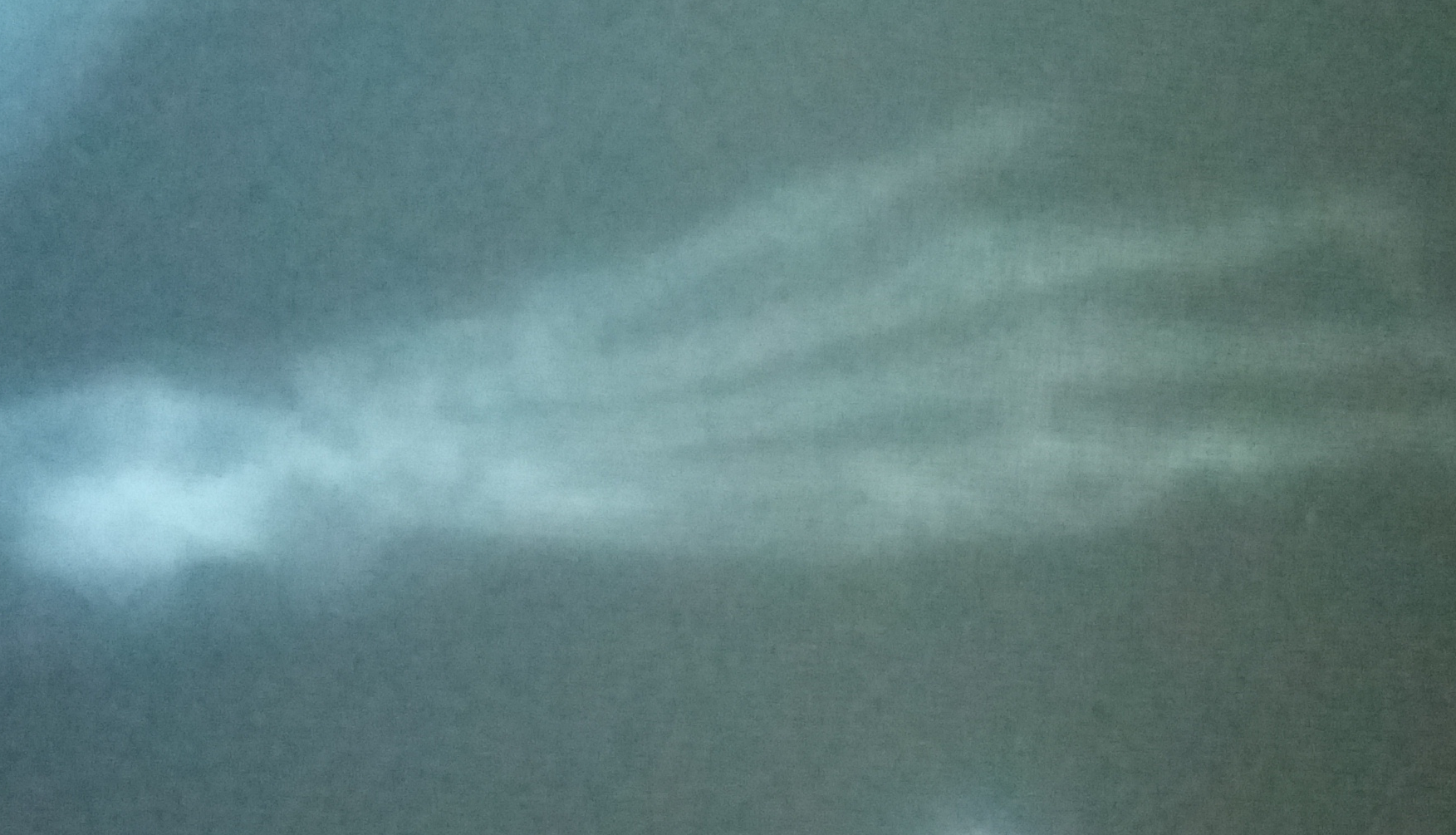  **(A) Normal** | 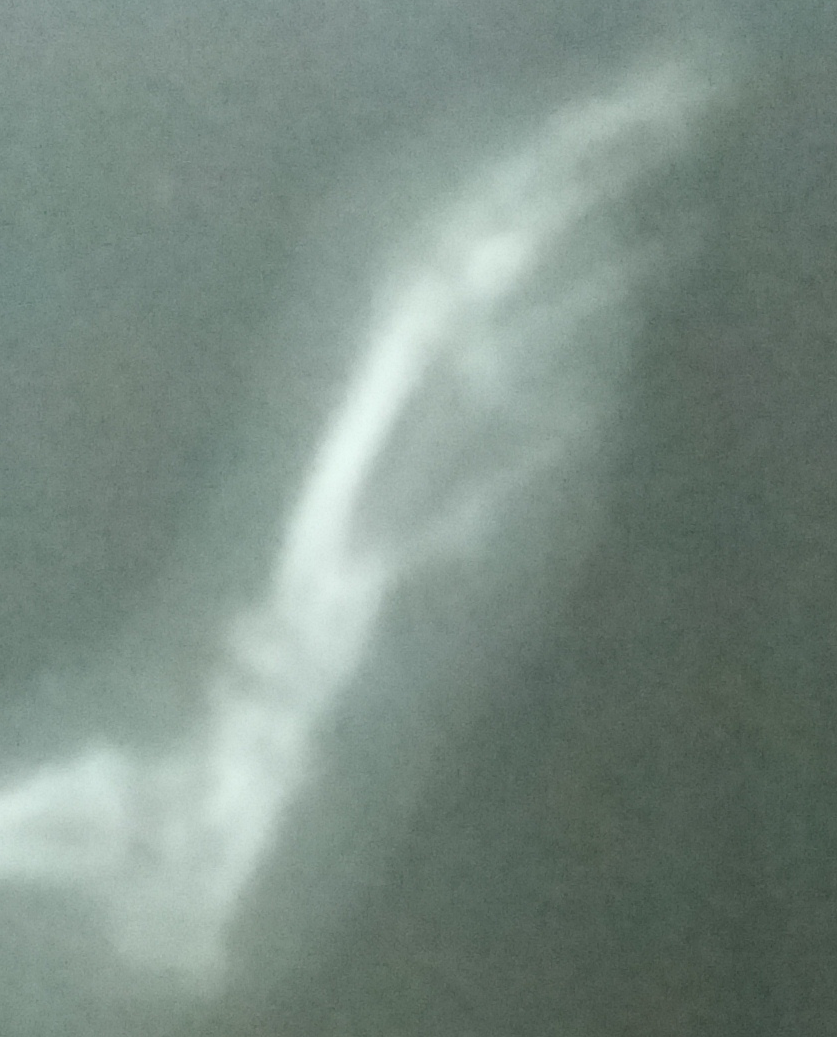  **(B) Arthritis** |
| --- | --- |
| 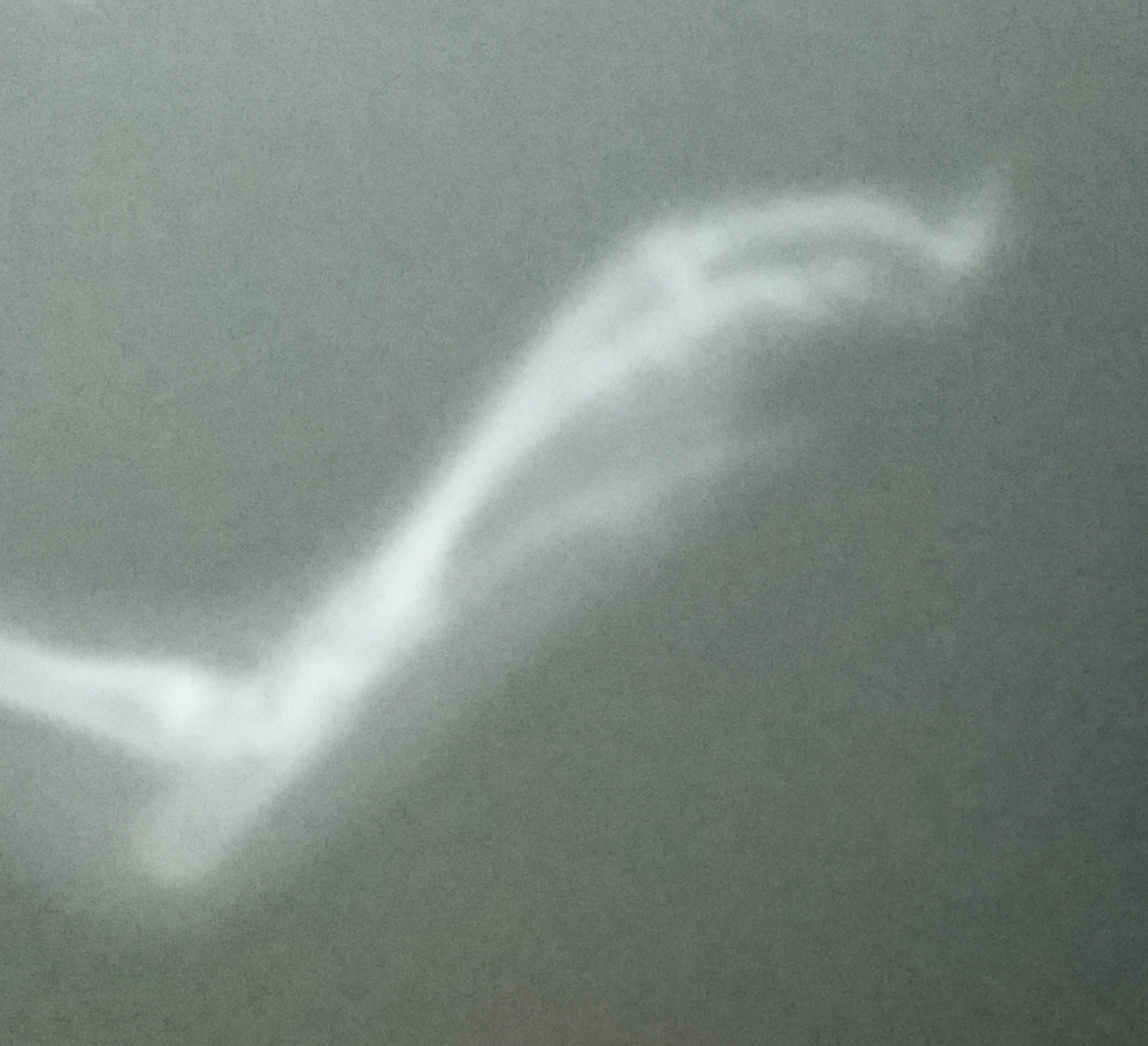**C) Test compound 9** | 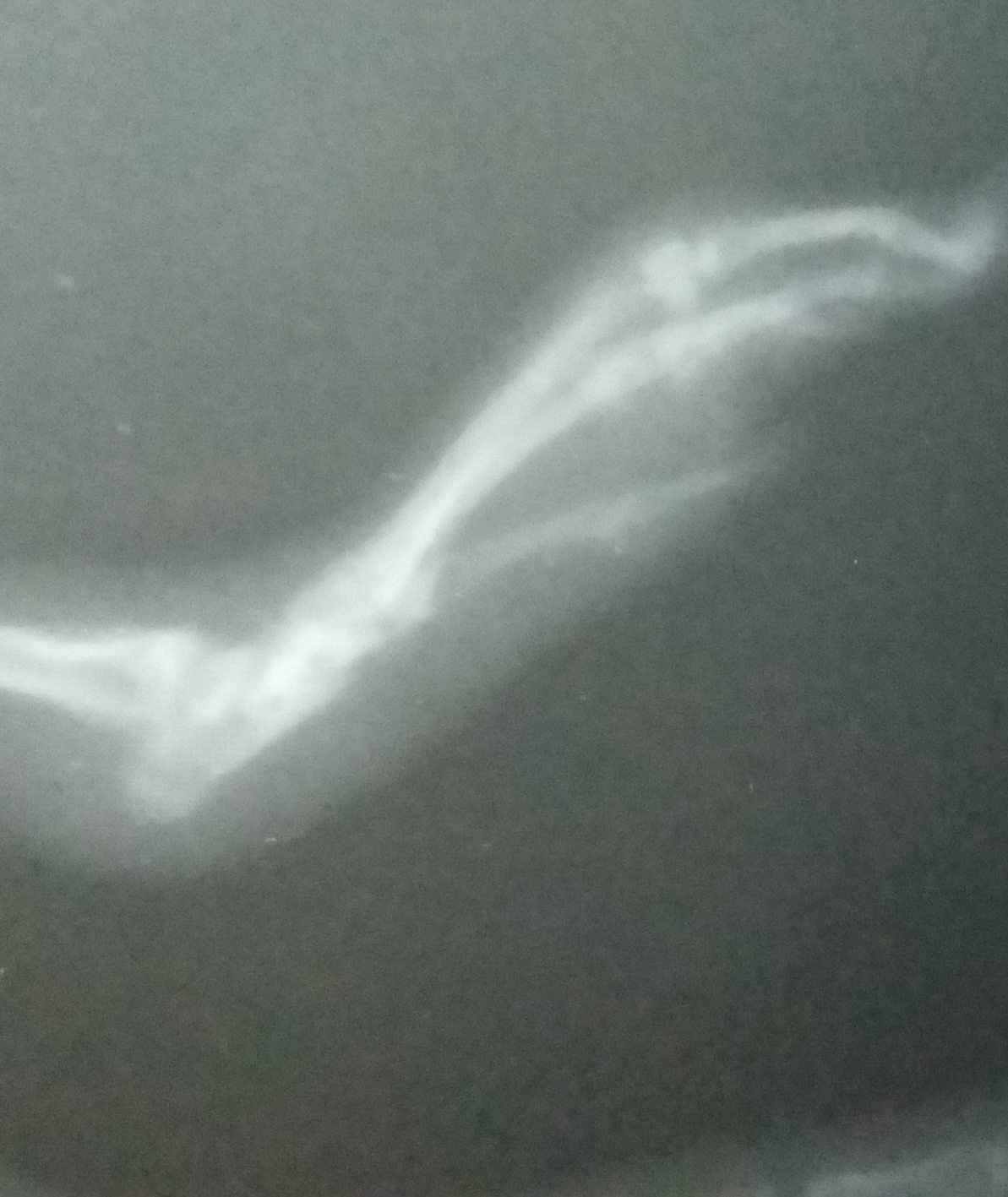**D) Test compound 15** |

**Figure S1.** Radiological characteristics of hind paws of arthritic rats.


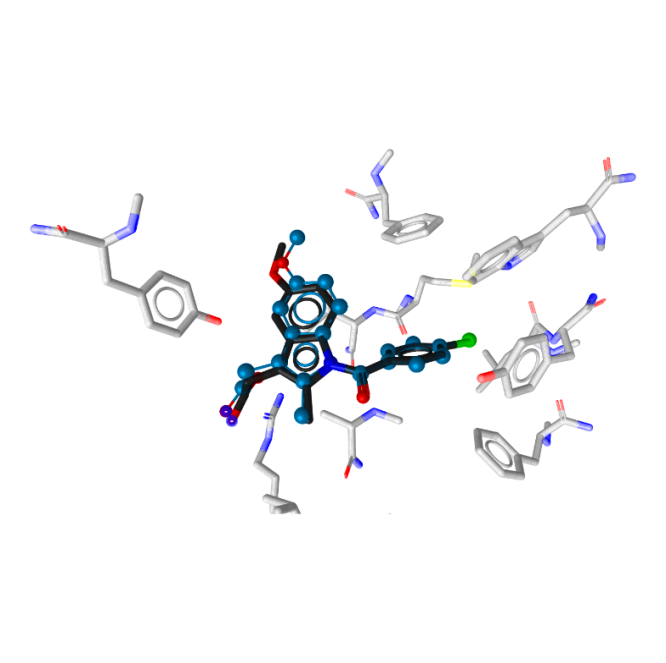

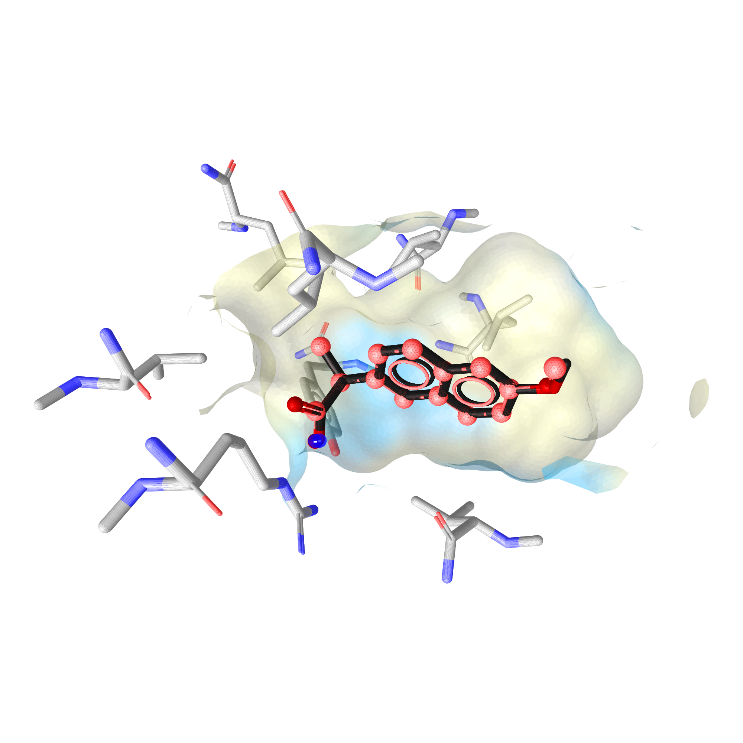


**Figure S2.** Software GOLD 5.2 generated binding mode of indomethacin (left) and naproxen (right) compared to their original co-crystalized conformations. **Left**: generated binding mode of indomethacin (blue, balls and sticks) in the PDB: 4COX compared to its experimental conformation (black sticks). **Right**: created binding mode of naproxen (pink, balls and sticks) in the PDB: 3NT1 compared to its co-crystal conformation (black sticks).
